# The Correlation between Maternal and Neonatal Vit D (25(OH)D) Levels in Greece: A Cross-Sectional Study

**DOI:** 10.3390/clinpract14030060

**Published:** 2024-04-26

**Authors:** Artemisia Kokkinari, Maria Dagla, Evangelia Antoniou, Aikaterini Lykeridou, Giannoula Kyrkou, Kostas Bagianos, Georgios Iatrakis

**Affiliations:** 1 Department of Midwifery, School of Health & Care Sciences, University of West Attica, 12243 Athens, Greece; mariadagla@uniwa.gr (M.D.); klyker@uniwa.gr (A.L.); ikirkou@uniwa.gr (G.K.); giatrakis@uniwa.gr (G.I.); 2 Biochemical Department of Tzaneio Piraeus General Hospital, 18536 Piraeus, Greece; kostasbagianos@gmail.com

**Keywords:** pregnancy, maternal 25(OH)D concentrations, neonatal 25(OH)D concentrations, fetal blood, Vit D deficiency, association between maternal and fetal blood or cord blood

## Abstract

Background: Few studies have correlated maternal and neonatal Vit D (25(OH)D) levels at birth in Greece. We investigated this potential association, taking into account the administration or not of low doses (400–800 IU) of prenatal Vit D supplements. Our study contributes evidence not only to the small amount of existing literature regarding the above correlation, but also to the topic of maternal and neonatal vitamin D deficiency (VDD) during pregnancy in Mediterranean countries, such as Greece. Methods: A cross-sectional study was conducted on 248 neonates and their mothers from September 2019 to January 2022. Blood samples of 25(OH)D were studied at the time of delivery. Frequency counts and percentages were registered, and logistic regression was used to investigate the independent factors associated with maternal Vit D status. The Chi-square test and the Pearson coefficient were used to demonstrate a possible association between maternal and neonatal 25(OH)D levels. Results: Our findings show a high prevalence of VDD in Greek women and their newborns at birth. This was observed not only in women who did not receive Vit D supplements, but also in all the study groups, especially in the autumn and winter months. We observed that mothers who received low doses (400–800 IU) of prenatal Vit D supplements increased both their own 25(OH)D concentrations and those of their newborns; however, the latter did not seem to be completely covered by the prenatal administration of Vit D because, although their 25(OH)D concentrations increased, they never reached sufficient 25(OH)D levels, unlike their mothers who reached sufficient concentrations. Conclusions: Overall, this study highlights the strong association between maternal and neonatal 25(OH)D concentrations at the end of gestation. However, neonates tended to show even lower 25(OH)D concentrations relative to maternal 25(OH)D concentrations. The same phenomenon was observed irrespective of the administration of Vit D supplements during pregnancy. Moreover, this is what was observed concerning the administration of formulations with 400–800 IU of Vit D, which the doctors in our clinic used in the present study. In any case, more clinical studies related to the administration of higher doses of Vit D supplementation to pregnant women would lead to more reliable conclusions. Without a doubt, the measurement of maternal vitamin D status during pregnancy provides opportunities for preventive and therapeutic interventions in the mother–infant pair.

## 1. Introduction

Vit D is a fat-soluble vitamin and is enzymatically converted in the liver to 25-hydroxyvitamin D (25(OH)D), which is the main form of circulation of Vit D [1]. 25(OH)D is further hydroxylated in the kidney to 1,25(OH)_2_D, which is its active form. Sun is the source of ultraviolet (UV) radiation, which stimulates the synthesis of Vit D3 in the skin from 7-dehydrocholesterol. Vit D synthesis efficiency is affected by age [2], skin pigmentation (melanin content) [2], season, weather, latitude, altitude, time of day, clothing style [3], exposed body surface, sun exposure habits and duration (especially on holiday), sunscreen use [3], and skin type [1]. Vit D production can be improved by higher dietary calcium intake, which has an effect on maintaining serum Vit D status by increasing the half-life of 25(OH)D [4]. The population recommendation in most countries to maintain sufficient Vit D concentrations throughout the year is a desirable combination of sun exposure, diet, consumption of fortified foods, and Vit D supplementation [4]. Even in the summer, Vit D production never occurs before 9 am and definitely stops after 4 pm [5]. 25(OH)D is used to assess serum Vit D status, as it reflects the sum of skin-produced Vit D and that obtained from diet and supplements [6].

Over the last few years, a number of agencies—the German Nutrition Society, the Dutch Health Ministry, the Nordic Council of Ministers (NORDEN), the UK Scientific Advisory Committee on Nutrition (SACN), the European Food Safety Authority (EFSA), and the European Society for Paediatric Gastroenterology Hepatology and Nutrition (ESPGHAN)—revised the definition of Vit D status, proposing different cut-off levels for sufficiency (>20 ng/mL or ≥30 ng/mL), insufficiency (10–20 ng/mL or 20–30 ng/mL), deficiency (10–20 ng/mL or <10 ng/mL), and severe deficiency (<10 ng/mL or <5 ng/mL) [7]. Several studies have associated maternal Vit D deficiency (VDD) with undesirable effects during pregnancy, impacting both the mother [8,9,10,11] and the newborn [10,11,12]. However, it is unclear whether low levels of Vit D are the causal factor of the undesirable effects or a marker of poor maternal health. Vit D metabolism in pregnancy shows striking differences compared with the non-pregnancy period [13]. Maternal 1,25(OH)_2_D3 increases two to three times more, in the first weeks of pregnancy, while maternal 25(OH)D can easily cross the placental barrier, unlike its active form, 1,25(OH)_2_D [13]. Thus, maternal 25(OH)D represents the primary source of fetal Vit D. However, some researchers have reported a decrease in 25(OH)D in late pregnancy [14]. Although the placenta has the ability to synthesize 1,25(OH)_2_D, this mainly takes place in the mother’s kidneys. Here, we should mention that fetal kidneys can also synthesize 1,25(OH)_2_D from 25(OH)D, with a likely complementary role [15]. Calcium is transferred from the mother to the fetus through the placenta. The placenta converts 25(OH)D to 1,25(OH)_2_D, which absorbs calcium from the maternal gut to meet the needs of the fetus [16]. Thus, it seems that during pregnancy, important changes occur regarding the mother’s Vit D concentration and calcium metabolism [16].

To date, most studies support that fetal 25(OH)D concentrations depend on maternal 25(OH)D concentrations. Although most observational studies indicate a significant linear relationship between maternal and neonatal 25(OH)D levels, this relationship has not been sufficiently studied in homogenous populations (same ethnicity, same country, same place, and shared sun exposure habits). This relationship has not been sufficiently studied in Mediterranean countries, such as Greece, which contributes to the synthesis of Vit D due to the sun, especially during the summer months. Most of the studies that have already been conducted and confirm this positive correlation concern the Asian population. A systematic review by Cashman et al. [17] reported that Afghanistan, Pakistan, India, Tunisia, Syria, the West Bank, Gaza, and Mongolia were classified as “hot spots” for very low 25(OH)D concentrations (<25–30 ng/mL) among women, pregnant women, or infants on the basis of having a prevalence in excess of 20%. This indicates that VDD is a major public health problem worldwide, even in countries with low latitudes, where it was generally assumed that UV radiation was adequate enough to prevent this deficiency. Nikolaidou et al. [18] tried to determine the actual situation in Greece. They evaluated serum 25(OH)D concentrations in 123 healthy mother–infant pairs from a public hospital in the sunny Athenian region. They observed that pregnant women who delivered in summer and autumn reported higher levels of 25(OH)D (18.9 [12.9–23.3] ng/mL) than those who delivered in winter and spring (14.6 [10.1–18.5] ng/mL). A strong correlation was also observed between maternal and infant 25(OH)D concentrations (r = 0.626, *p* < 0.001). They considered that the abundant sunlight exposure in Athens is not sufficient to prevent hypovitaminosis D, and pregnant women should be prescribed Vit D supplementation; moreover, they also suggested that the scientific community should consider Vit D supplementation of foods [18]. Karras et al. [13] determined serum (mothers) and umbilical cord (neonates) concentrations of all Vit D forms in a cohort study of 60 Caucasian pregnant women at term and their neonates in northern Greece. They showed that apart from being a reliable marker of Vit D maternal status, 25(OH)D comprises a significant parameter in predicting neonatal 25(OH)D3 concentrations, which constitutes the major neonatal Vit D form. Therefore, the few studies that have been carried out to date do not depict the true picture of the association between maternal and neonatal 25(OH)D levels in Greece.

With our study, we tried to contribute evidence to the paucity of literature on this topic. This study aims to evaluate any likely correlation between maternal and neonatal Vit D status at birth in Greece and evaluate the corresponding effects of Vit D intake, examining the samples of pregnant Greek women and their neonates who have benefited from the Mediterranean sun that influences Vit D synthesis. 

## 2. Materials and Methods

This study was designed to investigate whether there is a possible association between maternal and neonatal VDD at birth, taking into account the administration or not of prenatal Vit D supplements. Secondary outcome measures were to describe the variation in 25(OH)D levels during the seasons. To date, few studies have examined this relationship in the population of Greece, a Mediterranean country with high amounts of sunshine, which aids in the synthesis of 25(OH)D. 

**Figure 1:**
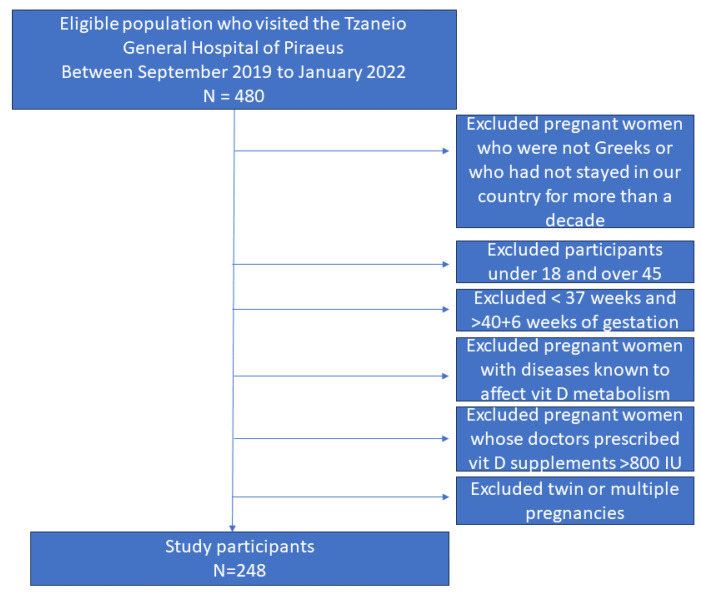
Flow diagram.

We conducted a cross-sectional study that enrolled a sample of 248 healthy, pregnant Greek women (>18 years old) giving birth at Tzaneio General Hospital of Piraeus. Piraeus is a port city in the region of Attica at latitude 37 56′ 50.82″ Ν. and, on average, receives around 3920 h of sunshine throughout the year [19]. Piraeus experiences the highest level of UV radiation in May, July, and August, when the maximum UV index can reach values of 10–10, which corresponds to the Very High category of sun exposure [19]. January, February, and December are in the Moderate exposure category. In these months, the maximum values of the UV index do not exceed 4 [19]. A total of 248 mother–infant pairs took part in our investigation, from September 2019 to January 2022. From our total sample of 248 pregnant patients, 221 (89.1%) of our sample are native Greeks and only 27 (10.9%) are Europeans who have been living permanently in Greece for more than a decade. This study involved all pregnant Greek women (or Europeans living in Greece for more than 10 years) who were cared for by doctors from our clinic throughout their pregnancy and gave birth at the Obstetrics and Gynecology clinic of the Tzaneio General Hospital of Piraeus. Their doctors recommended or not prenatal Vit D intake. Excluded from our study were pregnant women whose doctors we knew in advance would prescribe prenatal Vit D supplements > 800 IU (Figure 1). Pregnant women taking medications known to affect Vit D metabolism and Vit D supplementation, such as corticosteroids, antiepileptics, antituberculosis drugs, and antifungals, were also excluded (Figure 1). Exclusion criteria were also non-European pregnant patients as well as pregnant women who were not willing to participate in this study (Figure 1). Inclusion criteria for our study were all pregnant women giving birth in our clinic and who received or did not receive a regular dose of Vit D (400–800 IU/day) and calcium (500 mg/day) supplementation, according to the direction of their treating physicians. As previously stated, we recorded in advance what was prescribed by the doctors and the content of Vit D preparations in order to determine whether they would be included in our study. Under no circumstances did we modify doctor prescriptions, which were in accordance with the guidelines of the Greek Ministry of Health that states that doctors may administer Vit D supplementation to pregnant women as required [20]. 

A sample of four hundred people would have been ideal for this study; however, we settled for a sample of 250 people due to the hospital’s limited budget, which could not provide us with more than 500 kits to measure serum 25(OH)D. The study protocol was submitted to the hospital’s scientific board for evaluation and was subsequently approved (protocol number 5844 of 29 March 2018). The required approval was obtained before commencing this study. Data were collected from 248 healthy Greek mother–infant pairs. The procedure and its importance were explained to all the participants. All parents including the mother’s husbands signed an informed consent form. The laboratory information systems of the hospital were used with the agreement of the head of the Biochemistry Department, the hospital sector, and its administration. This study was carried out on the samples of the mother, at the scheduled laboratory test time, before delivery, and on the samples of the newborn immediately after birth. Circulating levels of 25(OH)D were studied in early-term pregnancies (after 37 + 0 to 38 + 6 weeks of gestation), but also in full-term pregnancies (after 39 + 0 to 40 + 6 weeks of gestation). Five milliliters (ml) of both the mother’s and neonate’s cord blood were collected with aseptic precautions, which were labeled and then transferred to the hospital laboratory to measure 25(OH)D levels. The analysis of maternal 25(OH)D levels was performed with a single test and was conducted only on the day of delivery. We did not analyze maternal 25(OH)D levels at the beginning or throughout pregnancy. All collected blood samples were processed within the collection day. The blood samples were analyzed in the laboratories of the Tzaneio General Hospital of Piraeus, using the ARCHITECT 25-OH Vitamin D 5P02 Reagent Kit. ARCHITECT is trademark of Abbott Laboratories in various jurisdictions (Abbott Ireland Diagnostics Division Lisnamuck, Longford/Co., Longford, Ireland). The ARCHITECT 25-OH Vitamin D assay is a chemiluminescent microparticle immunoassay (CMIA) for the quantitative determination of 25(OH)D vitamin in human serum and plasma. The ARCHITECT 25-OH Vitamin D assay is standardized against NIST SRM 2972 (National Institute of Standards and Technology Standard Reference Material 2972). The measuring interval of the ARCHITECT 25-OH Vitamin D assay is 3.4 to 155.9 ng/mL (8.5 to 389.8 nmol/L).

Each mother’s results were accompanied by a detailed medical history and a personal, standardized questionnaire. Both of these records provided us with information related to the presence or absence of factors influencing maternal 25(OH)D concentrations. The medical history included changes in weight and body mass index (BMI) (underweight (<18.5 Kg/m^2^), normal weight (18.5–24.9 Kg/m^2^), overweight (25–29.9 Kg/m^2^)), obesity (≥30) during pregnancy, weight gain in pregnancy (underweight, normal, or overweight), parity (primiparous, multiparous), pre-existing diseases, drug and supplement intake, gestational weeks, unwanted pregnancy outcomes, and term of delivery (normal or cesarean). The structured questionnaire contained details about demographic and phenotypic characteristics (age, height, weight, and parity), level of socioeconomic status (upper class, middle class, or lower class (the subjects themselves ranked their socioeconomic status)), dietary habits, sun exposure habits (20 min, 1 h, 2 h, >2 h, or no exposure), physical activity (no, yes), as well as possible complications of the present pregnancy and the taking or not of Vit D supplements. Physical activity was defined as bursts of activity of at least 15 min duration a day such as walking for 15 min. The mothers were divided into three age groups, 18–35 years (yr), 35–42 yr, and >42 yr. For each child, anthropometric and clinical data (gender, weight, height, and head circumference) were recorded. Neonate’s growth parameters were collected immediately after birth by midwives, using calibrated instruments. Instruments such as measuring tapes and weighing scales were all validated before being used for this study. 

The evaluation of maternal/neonatal Vit D concentrations was carried out according to the American Endocrine Society. Total serum 25-hydroxyVit D (D2 + D3) level (25(OH)D) was quantitatively determined and expressed as ng/mL. The following cut-offs were considered to qualitatively define the maternal Vit D status: (a) sufficiency (>30 ng/mL) [5]; (b) insufficiency (21–29 ng/mL) [5]; and (c) deficiency (<20 ng/mL) [5]. Here, perhaps there is another category d) of severe deficiency (<12 ng/mL) that could be added, based on a review by Amrein et al. [21] that reported on the current situation, worldwide, regarding 25(OH)D. The term VDD refers to serum 25(OH)D levels <30 ng/mL. According to the American Pediatric Endocrine Society, the newborns of the mothers of each category were also divided into (a) sufficiency (>30 ng/mL) [22]; (b) insufficiency (16–29 ng/mL); (c) deficiency (<15 ng/mL) [22]; and (d) severe deficiency (≤12.5 ng/mL) [23]. In our sample, the pregnant women were divided into two seasonal periods based on the sunshine of Greece. From a climatic point of view, based on the Hellenic National Meteorological Service (HNMS) 2020, we divided the year into two seasons: The warm, rainless, abundantly sunny season, which lasts from April to mid-October was defined as Group A, and the cold, rainy and sunless season, from mid-October to the end of March, was defined as Group B [24]. In this way, we quantified sunlight exposure. Mother–infant pairs were categorized into seasonal pairs (Group A and Group B). 

Data were processed using IBM SPSS Statistics 26 software and Microsoft EXCEL (v.2010; Microsoft Corp Redmond). The results are presented as means ± standard deviations (SD) or by frequencies and percentages, as appropriate. The normality of maternal Vit D levels was determined using the Kolmogorov–Smirnov test. Quantitative results of Vit D values in the mother–infant pair were transformed into qualitative variables assessing sufficiency, insufficiency, deficiency, and severe deficiency of maternal and neonatal concentrations. The Student’s *t*-test (for normally distributed data) or the Mann–Whitney U-test (for non-normally distributed data) were used for comparisons. Differences in frequencies were evaluated using the Chi-square test. The Chi-square test was used to find an association between maternal and neonatal 25(OH)D concentrations. The Pearson correlation coefficient was used to determine the strength of association between 25(OH)D Vit D concentrations in the pregnant mother–infant pair. The distribution of maternal demographic characteristics on the mothers’ 25(OH)D concentrations was compared between groups using the Kruskal–Wallis H-test. Multiple comparisons were adjusted with the Bonferroni calculation. Multivariate logistic regression analysis was applied to investigate the relationship. All *p*-values less than 0.05 (*p* < 0.05) were defined as statistically significant. 

## 3. Results

Most of the demographic characteristics of the 248 participants (pregnant women) are shown in (Table 1). Most pregnant women (78.62%) (195/248) belonged to the 18–35 age group. In the present study, a large number of the subjects (41.93%) (104/248) were from the middle socioeconomic class. The mean BMI was 24.93 Kg/m^2^. All measurements on neonates were carried out immediately after delivery. Their mean birth weight was 3163 ± 562 g, mean birth height was 50.57 ± 4.30 cm, and mean head circumference (HC) was 34.05 ± 1.60 cm. Both the maternal and neonatal serum 25(OH)D concentrations in blood samples were measured. Mean overall maternal 25(OH)D concentrations, including the standard deviation (SD), were 20.27 ± 11.6 ng/mL, with a confidence interval (CI) of 95% (95% CI: 1.18–1.45), while those of the neonates in umbilical cord blood were 14.47 ± 8.5 ng/mL (95%CI: 0.79–1.04), both approaching the boundaries of deficiency of 25(OH)D. The mean level of maternal and neonatal 25(OH)D concentrations in women who did not receive Vit D supplements throughout their pregnancy were approximately 16.92 ± 9,57 ng/mL (deficiency or insufficiency) and 12.64 ± 8.06 ng/mL (deficiency or insufficiency), respectively. The mean of maternal and neonatal 25(OH)D concentrations in women who received Vit D prenatally were 26.92 ± 12.43 ng/mL (insufficiency or sufficiency) and 18.10 ± 8.24 ng/mL (insufficiency), respectively. We carried out two independent *t*-tests, one for mothers and one for neonates. These tests were conducted to determine whether there was indeed a statistically significant difference between mean maternal 25(OH)D concentrations when mothers-to-be did or did not take prenatal vitamin D supplementation, and similarly, whether there was indeed a statistically significant difference in neonatal 25(OH)D concentrations in neonates whose mothers did or did not receive Vit D supplementation. In this way, we examined whether prenatal Vit D supplements had an effect on neonatal and maternal 25(OH)D concentrations. The dependent variable was either the maternal or neonatal 25(OH)D concentration while the independent variable was the categorical two-level variable, which was the intake or not of Vit D supplements. In both cases, given that the *p*-value [Sig (2-tailed)] was <0.05, we rejected the null hypothesis and concluded that for expectant mothers who took Vit D supplements and their respective neonates, 25(OH)D mean sample concentrations indeed differed between them when compared with the mean sample 25(OH)D measurements of those expectant mothers who did not receive Vit D supplements and their respective neonates (Table 2). 

Based on our sample, the percentage of VDD, including deficiency (<20 ng/mL) and severe deficiency (<12 ng/mL) of 25(OH)D, in mothers in Greece was found to be 58% (143/248). If we add insufficiency of 25(OH)D (<30 ng/mL), 25% (62/248), total maternal VDD increases to 83%, while only the remaining 17% of pregnant women samples exhibit 25(OH)D sufficiency. For newborns, VDD, including deficiency (<15 ng/mL) and severe deficiency (<12.5 ng/mL), was recorded at 66% (163/248). Similarly, if we add insufficiency of 25(OH)D (<30 ng/mL), 29% (72/248), total neonatal VDD increases to 95%, while only the remaining 5% (13/248) of neonates exhibit sufficiency (Table 3). 

**Figure 2:**
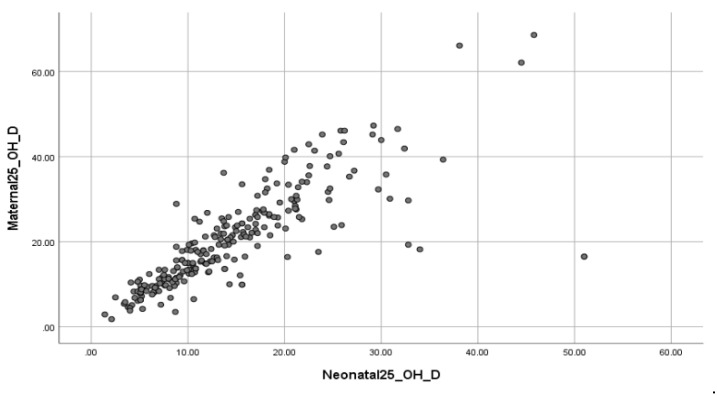
Scatter plot of correlation between maternal–neonatal 25(OH)D levels. The correlation between them is characterized as strong, as the nebula of observations is approximated by a straight line passing through the origin of the axes.

There was a statistically significant direct correlation between maternal and neonatal 25(OH)D concentrations (*p*-value/(*p*) of Chi-square test = 0 <0.001) (Table 4). Rejecting the null hypothesis of independence of variables, we inferred an association between maternal and neonatal 25(OH)D concentrations. In fact, the correlation between them is characterized as strong, based on the Pearson correlation coefficient = 0.8 (Table 5). This is also supported graphically, as the nebula of observations is approximated by a straight line passing through the origin of the axes (Figure 2). When mothers had a sufficiency of 25(OH)D (43/248), their newborns did not inherit the same trend but exhibited 25(OH)D insufficiency (33/248). The same phenomenon in which all newborns tend to have even lower 25(OH)D values in relation to maternal concentrations was observed even when women receive Vit D in their pregnancy. It was obvious that even when women had 25(OH)D sufficiency (26/83), benefiting from Vit D supplementation, prenatally, neonatal 25(OH)D levels were insufficient (<30 ng/mL) in 73% of cases (Table 6). In our study, 83/248 pregnant women received prenatal Vit D supplements while 165/248 received no supplement of any kind. For those who received Vit D supplements during their pregnancy, newborn 25(OH)D concentrations continued to follow their mother’s concentrations of 25(OH)D in exactly the same way as mentioned above. When mothers had a severe deficiency of 25(OH)D in their serum (8/83), their newborns, 87.5% (7/8), also had a severe deficiency of 25(OH)D. When mothers had a deficiency of 25(OH)D (17/83), their neonates, 59% (10/17), had even more severe deficiency and 29% (5/17) deficiency of 25(OH)D. When mothers had insufficiency of 25(OH)D levels (32/83) in their neonates, 50% (16/32) had 25(OH)D insufficiency. Finally, pregnant women with 25(OH)D sufficiency (26/83) gave birth to neonates with insufficiency of 25(OH)D in 73% (19/26) of the cases. 

On average, in the winter period, both the maternal and neonatal 25(OH)D mean values indicated a deficiency of 25(OH)D, 16.96 ± 9.6 ng/mL and 12.87 ± 8.2 ng/mL, respectively (Table 7). However, Group A had mean values that show an insufficiency, with the maternal mean value at 24.22 ± 12.57 ng/mL and the neonatal mean value at 16.37 ± 8.55 ng/mL (Table 7). The percentage of VDD and insufficiency of 25(OH)D (<30 ng/mL) of our total number of samples per seasonal period was 75% for mothers (85/113) and 92% for neonates (105/113) in the summer period (Group A). Similarly, in the winter period (Group B), this percentage was 89% (120/135) for mothers and 96% (130/135) for neonates. 

Maternal 25(OH)D concentrations were associated with parity (*p* = 0.004), smoking (*p* = 0.002), hours of sun exposure (*p* = 0.014), seasonal birth group (*p* < 0.001), and marginally with maternal age (*p* = 0.052). Socioeconomic status (upper class, middle class, or lower class) and type of delivery (normal or cesarean section) were *p* = 0.202 and *p* = 0.566, respectively, whereas weight gain (*p* = 0.088), physical activity (0.508), and BMI *(p* = 0.722) was not statistically significant at *p* < 0.05 level via Chi-square analysis (Table 1).

## 4. Discussion

The results of the present study provide a novel insight into the association of maternal and neonatal 25(OH)D concentrations at birth in the Mediterranean country of Greece, where conditions for the synthesis of Vit D are favorable due to sun exposure. Furthermore, our investigation allowed us to analyze the status of 25(OH)D and thus the prevalence of VDD at birth in Greece. To date, most observational studies have been carried out in Asian countries and although they recognize the association between maternal and neonatal 25(OH)D concentrations and the possibility that maternal VDD is a predisposing risk factor (RF) for neonatal VDD, more clinical studies are needed in Greece in order to reveal any association. 

Some of the main strengths of our study are that it was an investigation with a large number of samples that added evidence to both maternal and neonatal VDD for Greece, which until now had been minimally studied. It is worth noting that, as previously noted, most other studies were conducted in Asian countries. Our study was a cross-sectional study that concerned only the Greek population or people who have resided permanently in Greece for more than ten years and have obviously benefited from the Mediterranean sun in the synthesis of Vit D. Our study highlighted a high prevalence of VDD in Greece (Table 3) and a strong association between maternal and neonatal 25(OH)D concentrations (Table 4 and Table 5). These observations reflect previous reports of widespread VDD in Europe and the USA [21]. If this strong correlation between maternal and neonatal 25(OH)D concentrations (Table 5) is confirmed by future clinical studies, it would be of interest to develop a prenatal pregnancy selection program regarding maternal VDD early or during pregnancy that could predict neonatal VDD and its consequences at birth. Such a program would be useful in daily clinical practice. Healthcare professionals would be able to contribute to the appropriate management of maternal VDD throughout pregnancy in order to avoid neonatal VDD. When required, healthcare professionals would be able to prescribe Vit D supplementation or recommend Vit D from nutritional sources. To correct maternal VDD, it is deemed imperative to administer the correct dose of Vit D supplementation to the mother at the appropriate time during pregnancy. However, prospective clinical studies are required to support or reject our findings. Palacios and Gonzalez [25] described a high prevalence (>20%) of 25(OH)D < 30 nmol/L among pregnant women and infants in many countries, including South Asia and the Middle East. Up to 60% of women in India and 86% of infants in Iran had 25(OH)D < 30 nmol/L. Arora et al. [26] investigated Vit D status in mothers and their newborns in northern India. Their study showed a high prevalence of VDD in pregnant women. Moreover, cord blood 25(OH)D was strongly correlated with maternal serum values of 25(OH)D. A similar result was seen in a very recent study by Rabbani et al. [27] that showed a high prevalence of VDD in pregnant women and their newborns and a strong positive association between maternal and newborn 25(OH)D levels. Another recent study by Ghafarzadeh et al. [28] appeared to concur with the aforementioned studies that the mean concentration of Vit D in pregnant women and infants is low and is directly correlated with umbilical cord blood Vit D levels. The study by Esmeraldo et al. [29] also showed a strong positive correlation between maternal and neonatal 25(OH)D concentrations, with higher values in newborns. The highest 25(OH)D concentrations were found in small for gestational age (SGA) infants at term age. They speculated that their findings may have been influenced by newborn body composition. Several randomized controlled trials (RCTs) have shown that prenatal Vit D supplementation contributes to increased neonatal cord 25(OH)D concentrations [30]. Lee et al. [31] investigated the association between maternal and neonatal 25(OH)D concentrations in 40 healthy, predominantly black, mother–infant pairs who received a daily prenatal multivitamin. In this study, they defined VDD for values <30 nmol/L. It was found that in 50% of mothers and 65% of their newborn infants who were more deficient than their mothers, there was a positive correlation between maternal and infant 25(OH)D plasma concentrations. Therefore, they considered that maternal VDD may be an important RF for the development of rickets in children. The study by Tsetendaba et al. [32] found a strong correlation between the amount of Vit D in the mother and in the newborn. They hypothesized that the mother’s Vit D intake was related to Vit D levels in the mother’s blood. However, it is difficult to draw conclusions on the need for Vit D intake during pregnancy due to the heterogeneous design of the studies in terms of the length of VDD, regimen of Vit D intake, and other potential confounding factors. Maghbooli et al. [33] confirmed the positive correlation between maternal and neonatal 25(OH)D concentrations in blood samples of 552 Iranian pregnant women and their newborns. In fact, in this study, the prevalence of newborn VDD was much higher compared with maternal 25(OH)D levels (66.8% and 93.3%, respectively) than in the study by Lee et al. [31]. However, even though the most recent study by Arora et al. [26] found a high correlation between maternal and neonatal 25(OH)D concentrations, umbilical cord 25(OH)D levels were slightly lower.

The present study, as far as we know, is the third one conducted in Greece. The first study was conducted by Karras et al. [13], with only 60 mothers and their newborns. Karras et al. [13] showed that, apart from being a reliable marker of Vit D maternal status, 25(OH)D comprises a significant parameter in predicting neonatal 25(OH)D3 concentrations, which constitutes the major neonatal Vit D form. Our study selected a larger sample size of 248 pregnant women and their neonates in order to contribute evidence to the small amount of existing literature; however, our results demonstrate the same findings in that there is a strong correlation between maternal and neonatal 25(OH)D concentrations. Consequently, previous findings are now more reliable. In order to clarify the association, in our investigation we divided our sample into two seasonal periods (Table 7), with or without sunlight, taking into account the administration or not of prenatal Vit D supplements. The seasonal effect appeared to be significant, both on maternal and neonatal 25(OH)D levels. We found that in the winter period, both the maternal and neonatal 25(OH)D concentrations indicated a deficiency of 25(OH)D (Table 7). Our study revealed that indeed winter months are considered risk factors (RF) for both maternal and neonatal VDD at birth. A higher prevalence of VDD at birth was observed, not only in those women who did not receive Vit D supplements, as mentioned above, but also especially, in the autumn and winter months from October 15 to the end of March (Group B) (Table 7). As revealed, especially during the winter months, newborns are a group at a higher risk than their pregnant mothers of developing severe 25(OH)D deficiency, especially when the mother does not take a Vit D supplement during pregnancy. At least this is what was seen with regard to the administration of formulations with 400–800 IU of Vit D, which the doctors in our clinic used in the present study. In the future, it is possible that administering higher doses of Vit D supplementation to pregnant women, especially in the winter months, to correct VDD would lead to more reliable conclusions about the potential, additional benefit in improving both fetal and neonatal 25(OH)D concentrations, given the correlation of maternal and neonatal 25(OH)D levels. In conclusion, besides administering supplemental Vit D, which seems to increase maternal and neonatal 25(OH)D concentrations (Table 6), the season of gestation must also be taken into account so that health professionals may recommend the optimum dosage regimen for pregnant women, carefully considering that in a Mediterranean country like Greece, the population seems to achieve higher 25(OH)D levels in the summer period. Conversely, in winter, it is more necessary for Greek women to bolster their Vit D through supplements as they exhibit higher VDD levels due to lower sun exposure. The second study was conducted by Nikolaidou et al. [18], who tried to determine the actual situation in Greece. They evaluated serum 25(OH)D concentrations in 123 healthy mother–infant pairs from a public hospital in the sunny Athenian region but none of the mothers had been prescribed Vit D supplements as in our study. Our findings concurred with previous studies in Greece about a definite high prevalence of VDD in women and in their newborns at birth (Table 3). It is notable that in a Mediterranean country, like Greece, our study showed that the total VDD, adding insufficiency, deficiency, and severe deficiency, approached 83% in mothers and an impressive 95% in newborns. We also found, like others [13,18], a strong positive correlation between maternal and cord blood 25(OH)D plasma concentrations (Table 5). The correlation between maternal and neonatal 25(OH)D concentrations is characterized as strong based on the Pearson correlation coefficient = 0.8 (Table 4). At this point, we have to mention that Lee et al. [31] and Maghbooli et al. [33] observed that infants had lower 25(OH)D concentrations compared with their mothers (Table 6) in an Asian population, which we likewise observed in Greece. Taking into account the fact and supported by the findings of our study, Vit D intake of 400–800 IU seemed to help the mother–infant pair, mainly by increasing the mean of the 25(OH)D maternal–infant concentrations. However, even though pregnant women took Vit D supplementation, we observed the same phenomenon; the prevalence of newborn VDD was much higher compared with maternal 25(OH)D levels. We hypothesized that newborns do not seem to be completely covered by the administration of exogenous Vit D intake of their mothers, at least with regard to the administration of formulations with 400–800 IU of Vit D, which the doctors in our clinic used in the present study. Although neonatal 25(OH)D concentrations increased after prenatal Vit D intake of 400–800 IU/day of their mothers, they never reached sufficient levels of 25(OH)D, unlike their mothers who reached them. This may be another indication that employing higher doses of Vit D supplements in pregnant women may well result in a greater increase in maternal–neonatal concentrations. Our findings are inconsistent with the study by Esmeraldo et al. [29] who recorded higher levels of 25(OH)D in newborns compared with maternal levels, while we observed the opposite. However, their findings referred to SGA infants. It is probable, as they mentioned, that their findings may have been influenced by newborn body composition.

To date, Vit D supplementation has not necessarily been part of prenatal care programs. Due to the limited evidence currently available to directly assess the benefits and drawbacks of Vit D supplementation, the use of this intervention during pregnancy as part of routine antenatal care is also not recommended (conditional recommendation). In the case of documented deficiency of 25(OH)D, Vit D supplements may be given at the current RNI (200 IU) per day as recommended by the World Health Organization (WHO)/FAO [34] or according to national guidelines. The US National Academy of Sciences states 400 IU/day as the minimum recommended dietary reference intake for prevention for newborns and infants up to 12 months of age, and 600 IU/day for pregnant and lactating women during pregnancy. Although, in the past years, several researchers from around the world supported revised guidelines for a higher amount of Vit D during pregnancy and lactation [35,36], newer research such as that of May Loong Tan et al. [37] considered that maternal higher dose supplementation (≥4000 IU/day) produced similar infant 25(OH)D levels as infant supplementation of 400 IU/day. The Endocrine Society Clinical Practice Guideline suggests that pregnant and lactating women require at least 600 IU per day of Vit D and recognize that at least 1500–2000 IU per day of Vit D may be needed to maintain a blood level of 25(OH)D above 30 ng/mL [5]. According to the article of Jouanne et al. [38], the current recommended Vit D intake amounts are set at 400–600 IU/day [38]. However, this intake is often insufficient, particularly during the third trimester and during the months of low sunshine [38]. When Vit D is only administered in the third trimester, 1000 IU/day is then necessary to obtain concentrations of 25(OH)D within normal limits in the mother and in the cord blood. The same results can be obtained with a single dose of 2000 IU administered at the start of the seventh month [38]. According to the WHO, for pregnant women with suspected VDD, Vit D supplements may be given at the current recommended nutrient intake of 200 IU/day [39]. This may include women in populations where sun exposure is limited [39]. In Greece, most public hospitals recommend Vit D supplementation to newborns regardless of whether they are breastfed. All infants born in our hospital’s maternity clinic are advised to start receiving a Vit D supplement containing 400 IU of Vit D within days of birth. Information related to the common practice of administering Vit D supplements to infants was corroborated and confirmed by the Director of the Pediatric Clinic of Tzaneiο General Hospital, Piraeus, Dr. George Triantafillidis. For infants, Vit D is included in most nonprescription infant multiVit drops [39]. This is a common practice in most public hospitals in Greece. In Greece, infant drops are available that contain only Vit D. In our study, while the administered dose of 400–800 IU/day seems to cover pregnant women, elevating 25(OH)D concentrations, it does not particularly cover their newborns, who seem unaffected and continue to have VDD. From our study, it appeared that the epidermal composition and dietary intake of Vit D in the susceptible population of pregnant women are insufficient, even in our own sunny country of Greece. It must be mentioned that during the winter months, the prevalence of maternal VDD showed elevated values (75% VDD (Group A) vs. 88% VDD (Group B) (*p* = 0.00)), while conversely, the prevalence of neonatal VDD was seemingly unaffected (92%VDD (Group A) vs. (96%VDD Group B) (*p* = 0.001)) by the lack of sunlight. Possibly, neonates are not particularly affected by seasonal fluctuations and through some mechanism maintain a steady minimum level of 25(OH)D, irrespective of the winter or summer periods that markedly influence their mothers. 

On the other hand, some of the disadvantages of our study are that a sample of four hundred people would have been ideal to reduce the statistical error; however, we settled for a sample of 248 people due to the limited deliveries of our clinic but also because of the limited budget of the hospital, which could not provide us with more than 500 kits for measuring serum 25(OH)D. Moreover, due to the nature of this study, some limitations should be considered. Because our study is a cross-sectional one, no causal relationships can be drawn. Another limitation that might have biased our results was the fact that the analysis of maternal and neonatal 25(OH)D levels was a single test that was only conducted on the day of delivery; no test was conducted at the start or during pregnancy. As such, our results do not imply that the recorded levels are typical for the entire period of pregnancy. Another limitation of our study is that we did not measure calcium and PTH levels in maternal–neonatal blood. Thus, the results should be interpreted with caution.

Since pregnant women in Greece are a high-risk group for VDD, these data justify the need for a program designed as a prenatal precursor to the healthcare model. Such a program could include preventive intervention elements that address specific activities in order to provide vitamin D sufficiency to pregnant women in support of optimal maternal and newborn health in pregnancy. In particular, pregnant women in Greece may need screening, especially during the winter months, prenatally, and at the beginning of gestation. Therefore, it is considered necessary to study maternal and neonatal 25(OH)D co-dependence and interdependence during pregnancy, in different weeks of pregnancy, and not only at birth, taking into account the mother’s BMI, before and at the end of pregnancy, the frequency and time of sample taking, and the interpretation of the results in Greece, given the diversity in sunshine.

## 5. Conclusions

Within the scope of this cross-sectional study, a high prevalence of maternal and neonatal VDD status was observed in healthy pregnant Greek women at term at the specific time of childbirth. Given the strong association between maternal and neonatal 25(OH)D concentrations that we found in our investigation, mothers who received low doses of prenatal Vit D supplements (400–800 IU) increased both their own 25(OH)D concentrations and those of their newborns. Although prenatal Vit D supplements contributed to increases in both maternal and neonatal 25(OH)D concentrations, we observed that the prevalence of newborn VDD was much higher than maternal 25(OH)D levels. This was observed with regard to the administration of formulations with 400–800 IU of Vit D, which the doctors in our clinic used in the present study. Our study revealed that, indeed, winter months are considered risk factors (RFs) only for maternal but not for neonatal VDD at birth. Newborns do not seem to be particularly affected by seasonal variations. On the other hand, newborns are at a higher risk than their mothers of developing severe 25(OH)D deficiency, especially when the mother does not take a Vit D supplement during pregnancy. The magnitude of VDD suggests the need for public health intervention and planning strategies for the prevention of VDD. Therefore, the Greek government and health professionals should attach importance to the high prevalence of maternal VDD in pregnancy and take its prevention as a priority, as our findings suggest that maternal 25(OH)D levels play an important role in influencing neonatal Vit D status. More awareness of VDD, more screening, and more food fortification with Vit D may support important positive changes in both maternal and neonatal VDD status. Undoubtedly, Vit D supplementation can lead to an increase in both maternal and neonatal Vit D status in pregnancy. Health professionals should be encouraged to change the trends of individuals with increased BMI and decreased physical activity, which may compromise Vit D status. In conclusion, additional research is warranted to Investigate the underlying mechanisms and therapeutic implications of these findings. Further prospective studies in Greece with higher administered doses of Vit D supplementation in pregnant women could guide us to possible additional benefits in neonatal umbilical cord 25(OH)D concentrations.

## Figures and Tables

**Table 1:** The association between maternal 25(OH)D concentrations and demographic and obstetrical characteristics. N represents the sample size from each category.

Maternal Characteristic	25(OH)D Status (ng/mL)					X^2^/*p*-Value
	Severe Deficiency *n* = 70	Deficiency *n* = 73	Insufficiency *n* = 62	Sufficiency *n* = 43	Total *n* = 248	
Μaternal age [Years (Yrs)]						0.052
18–35	62 (88.57%)	53 (72.60%)	50 (80.64%)	30 (69.76%)	195 (78.62%)	
35–42	7 (10.01%)	16 (21.91%)	9 (14.51%)	13 (30.23%)	45 (18.14%)	
>42	1 (01.42%)	4 (05.47%)	3 (04.83%)	0 (0.00%)	8 (03.22%)	
Βody mass index (BMI) [Kg/m2]						0.722
Underweight (<18.5)	6 (08.57%)	5 (06.84%)	5 (08.06%)	4 (09.30%)	20 (08.06%)	
Healthy weight (18.5–24.9)	36 (51.42%)	42 (57.53%)	38 (61.29%)	23 (53.48%)	139 (56.04%)	
Overweight (25–29.9)	8 (11.42%)	10 (13.69%)	6 (09.67%)	8 (18.60%)	32 (12.90%)	
Obesity (≥30)	19 (27.14%)	16 (21.91%)	13 (20.96%)	7 (16.27%)	55 (22.17%)	
Groups						0
Group A	20 (28.57%)	27 (36.98%)	38 (61.29%)	28 (65.11%)	113 (45.56%)	
Group B	50 (71.42%)	46 (63.01%)	24 (38.70%)	15 (34.88%)	135 (54.43%)	
Parity						0.004
Primiparous	44 (62.85%)	32 (43.83%)	39 (62.90%)	23 (53.48%)	138 (55.64%)	
Multiparous	26 (37.14%)	41 (56.16%)	23 (37.09%)	20 (46.51%)	68 (27.41%)	
Socioeconomic status						0.202
Upper class	21 (30.01%)	15 (20.54%)	9 (14.51%)	16 (37.20%)	61 (24.59%)	
Middle class	47 (67.14%)	54 (73.97%)	53 (85.48%)	27 (62.79%)	181 (72.98%)	
Lower class	2 (02.85%)	4 (05.47%)	0 (0.00%)	0 (0.00%)	6 (02.41%)	
Weight gain in pregnancy						0.088
Normal	24 (34.28%)	35 (47.94%)	29 (46.77%)	13 (30.23%)	101 (40.72%)	
Underweight	23 (32.85%)	20 (27.39%)	9 (14.51%)	16 (37.20%)	68 (27.41%)	
Overweight	22 (31.42%)	20 (27.39%)	24 (38.70%)	13 (30.23%)	79 (31.85%)	
Type of delivery						0.566
Normal	31 (44.28%)	25 (34.24%)	27 (43.54%)	19 (44.1%)	102 (41.12%)	
Cesarean	39 (55.71%)	48 (65.75%)	35 (56.45%)	24 (55.81%)	146 (58.87%)	
Direct sun exposure						0.014
20 min	19 (27.14%)	26 (35.61%)	16 (25.80%)	14 (32.55%)	75 (30.24%)	
1 h	19 (27.14%)	16 (21.91%)	21 (33.87%)	21 (48.83%)	77 (31.04%)	
2 h	7 (0.1%)	5 (06.84%)	3 (04.83%)	3 (06.97%)	18 (07.25%)	
>2 h	10 (14.28%)	20 (27.3%)	11 (17.74%)	4 (09.30%)	45 (18.14%)	
No exposure	15 (21.42%)	6 (08.21%)	11 (17.74%)	1 (02.32%)	33 (13.30%)	
Activity						0.508
NO	66 (94.28%)	67 (91.78%)	56 (90.32%)	37 (86.04%)	226 (91.12%)	
YES	4 (05.71%)	6 (08.21%)	6 (09.67%)	6 (13.95%)	22 (08.87%)	
Smoking						0.002
NO	29 (41.42%)	48 (65.75%)	42 (67.74%)	30 (69.76%)	149 (60.08%)	
YES	41 (58.57%)	25 (34.24%)	20 (32.25%)	13 (30.23%)	99 (39.91%)	
Total	70	73	62	43	248	

There is statistical significance when the *p*-value ≤ 0.05.

**Table 2:** Investigation, using the independent samples *t*-test, of the existence of a statistically significant difference between the mean values of maternal or neonatal 25(OH)D concentrations when expectant mothers took or did not take prenatal vitamin D supplementation.

Independent Samples *t*-Test										
		F	Sig.	t	df	Sig. (2-Tailed)	Mean Difference	Std. Error Difference	95% Confidence Interval of the Difference	
									Lower	Upper
Maternal 25(OH)D	Equal variances assumed	3474	64	−7002	246	*p* < 0.0001	−999,625	142,770	−1,280,833	−718,417
	Equal variances not assumed			−6431	132,332	*p* < 0.0001	−999,625	155,444	−1,307,100	−692,149
Neonatal 25(OH)D	Equal variances assumed	270	604	−5.002	246	*p* < 0.0001	−546.785	109.306	−762.079	−331.491
	Equal variances not assumed			−4.966	161.263	*p* < 0.0001	−546.785	110.107	−764.222	−329.348

**Table 3:** The evaluation of maternal/neonatal 25(OH)D concentrations. N represents the sample size.

Maternal 25(OH)D and Neonatal 25(OH)D Cross-Tabulation						
		Neonatal 25(OH)D				
		Severe Deficiency	Deficiency	Insufficiency	Sufficiency	Total
Maternal 25(OH)D	Severe deficiency	67	3	0	0	70
	Deficiency	48	17	4	4	73
	Insufficiency	5	21	35	1	62
	Sufficiency	0	2	33	8	43
Total	N	120	43	72	13	248

**Table 4:** Correlation with Chi-square test results of maternal–neonatal 25(OH)D concentrations.

Chi-Square Test			
	Value	df	Asymptotic Significance (2-Sided)
Pearson Chi-square	204.653	9	<0.001
Likelihood ratio	243.064	9	<0.001
Linear-by-linear association	151.838	1	<0.001
N of valid cases (n)	248		

**Table 5:** Correlation of maternal–neonatal 25(OH)D with Pearson coefficient.

Correlations			
	Maternal 25(OH)D		Neonatal 25(OH)D
Maternal 25(OH)D	Pearson correlation	1	0.800
	Sig. (2-tailed)		<0.001
	n	248	248
Neonatal 25(OH)D	Pearson correlation	0.800	1
	Sig. (2-tailed)	<0.001	
	n	248	248
If r = ±1, there is a perfect linear correlation. If −0.3 ≤ r ≤ −0.3 or 0.3 ≤ r < 0.5, there is no linear correlation. However, this does not mean that there is no other kind of correlation between the two variables. If −0.5 < r ≤ −0.3 or 0.3 ≤ r < 0.5, there is a weak linear correlation. If −0.7 < r ≤ −0.5 or 0.5 ≤ r < 0.7, there is an average linear correlation. If −0.8 < r ≤ −0.7 or 0.7 ≤ r < 0.8, there is a strong linear correlation. If −1 < r ≤ −0.8 or 0.8 ≤ r < 1, there is a very strong linear correlation.			

**Table 6:** Correlation between maternal–neonatal 25(OH)D concentration levels of pregnant women taking supplemental vitamin D.

Maternal 25(OH)D and Neonatal 25(OH) Cross-Tabulation						
	Neonatal 25(OH)D					
		Severe Deficiency Deficiency		Insufficiency	Sufficiency	Total
Maternal 25(OH)D	Severe deficiency	7	1	0	0	8
	Deficiency	10	5	1	1	17
	Insufficiency	2	14	16	0	32
	Sufficiency	0	1	19	6	26
Total (n)		19	21	36	7	83

**Table 7:** Prevalence of 25(OH)D maternal and neonatal concentrations in two seasonal periods.

Group A (Summer Period): April to Mid-October			
Group A			
Chi-Square Test			
	Value	df	Asymptotic Significance (2-Sided)
Pearson Chi-square	96.825	9	<0.001
Likelihood ratio	112.710	9	<0.001
Linear-by-linear association	72.511	1	<0.001
N of valid cases (n)	113		
**Group B (winter period): 15th October to End of March**			
**Group B**			
**Chi-Square Test**			
	**Value**	**df**	**Asymptotic Significance (2-Sided)**
Pearson Chi-square	120.096	9	<0.001
Likelihood ratio	128.987	9	<0.001
Linear-by-linear association	72.178	1	<0.001
N of valid cases (n)	135		
**Seasonal Periods (Group A and Group B) and Maternal 25(OH)D**			
**Chi-Square Test**			
	**Value**	**df**	**Asymptotic Significance (2-Sided)**
Pearson Chi-square	23.124	3	<0.001
Likelihood ratio	23.512	3	<0.001
Linear-by-linear association	21.253	1	<0.001
N of valid cases (n)	135		

## Data Availability

The data are not publicly available due to the Principle of Personal Data protection regulations but can be obtained upon a reasonable request to the corresponding author. Application number of Request to collect data: 7380/27 May 2019.
